# COVLIAS 3.0: cloud-based quantized hybrid UNet3+ deep learning for COVID-19 lesion detection in lung computed tomography

**DOI:** 10.3389/frai.2024.1304483

**Published:** 2024-06-28

**Authors:** Sushant Agarwal, Sanjay Saxena, Alessandro Carriero, Gian Luca Chabert, Gobinath Ravindran, Sudip Paul, John R. Laird, Deepak Garg, Mostafa Fatemi, Lopamudra Mohanty, Arun K. Dubey, Rajesh Singh, Mostafa M. Fouda, Narpinder Singh, Subbaram Naidu, Klaudija Viskovic, Melita Kukuljan, Manudeep K. Kalra, Luca Saba, Jasjit S. Suri

**Affiliations:** ^1^Advanced Knowledge Engineering Center, GBTI, Roseville, CA, United States; ^2^Department of CSE, PSIT, Kanpur, India; ^3^Department of CSE, IIIT, Bhubaneswar, India; ^4^Department of Radiology, “Maggiore della Carità” Hospital, University of Piemonte Orientale (UPO), Novara, Italy; ^5^Department of Radiology, A.O.U., Cagliari, Italy; ^6^Department of Civil Engineering, SR University, Warangal, Telangana, India; ^7^Department of Biomedical Engineering, NEHU, Shillong, India; ^8^Heart and Vascular Institute, Adventist Health St. Helena, St. Helena, CA, United States; ^9^School of CS and AI, SR University, Warangal, Telangana, India; ^10^Department of Physiology and Biomedical Engineering, Mayo Clinic College of Medicine and Science, Rochester, MN, United States; ^11^Department of Computer Science, ABES Engineering College, Ghaziabad, UP, India; ^12^Department of Computer science, Bennett University, Greater Noida, UP, India; ^13^Bharati Vidyapeeth’s College of Engineering, New Delhi, India; ^14^Division of Research and Innovation, Uttaranchal Institute of Technology, Uttaranchal University, Dehradun, India; ^15^Department of ECE, Idaho State University, Pocatello, ID, United States; ^16^Department of Food Science and Technology, Graphic Era Deemed to be University, Dehradun, India; ^17^Department of EE, University of Minnesota, Duluth, MN, United States; ^18^University Hospital for Infectious Diseases, Zagreb, Croatia; ^19^Department of Interventional and Diagnostic Radiology, Clinical Hospital Center Rijeka, Rijeka, Croatia; ^20^Department of Radiology, Massachusetts General Hospital, Boston, MA, United States; ^21^Department of Computer Science, Graphic Era Deemed to Be University, Dehradun, Uttarakhand, India; ^22^Symbiosis Institute of Technology, Nagpur Campus, Symbiosis International (Deemed University), Pune, India; ^23^Stroke and Monitoring Division, AtheroPoint LLC, Roseville, CA, United States

**Keywords:** COVID-19, computed tomography, COVID lesions, glass ground opacities, segmentation, hybrid deep learning, quantization

## Abstract

**Background and novelty:**

When RT-PCR is ineffective in early diagnosis and understanding of COVID-19 severity, Computed Tomography (CT) scans are needed for COVID diagnosis, especially in patients having high ground-glass opacities, consolidations, and crazy paving. Radiologists find the manual method for lesion detection in CT very challenging and tedious. Previously solo deep learning (SDL) was tried but they had low to moderate-level performance. This study presents two new cloud-based quantized deep learning UNet3+ hybrid (HDL) models, which incorporated full-scale skip connections to enhance and improve the detections.

**Methodology:**

Annotations from expert radiologists were used to train one SDL (UNet3+), and two HDL models, namely, VGG-UNet3+ and ResNet-UNet3+. For accuracy, 5-fold cross-validation protocols, training on 3,500 CT scans, and testing on unseen 500 CT scans were adopted in the cloud framework. Two kinds of loss functions were used: Dice Similarity (DS) and binary cross-entropy (BCE). Performance was evaluated using (i) Area error, (ii) DS, (iii) Jaccard Index, (iii) Bland–Altman, and (iv) Correlation plots.

**Results:**

Among the two HDL models, ResNet-UNet3+ was superior to UNet3+ by 17 and 10% for Dice and BCE loss. The models were further compressed using quantization showing a percentage size reduction of 66.76, 36.64, and 46.23%, respectively, for UNet3+, VGG-UNet3+, and ResNet-UNet3+. Its stability and reliability were proved by statistical tests such as the Mann–Whitney, Paired *t*-Test, Wilcoxon test, and Friedman test all of which had a *p* < 0.001.

**Conclusion:**

Full-scale skip connections of UNet3+ with VGG and ResNet in HDL framework proved the hypothesis showing powerful results improving the detection accuracy of COVID-19.

## 1 Introduction

SARS-CoV-2 is an infectious illness and a severe acute respiratory syndrome coronavirus 2 that has affected nearly 677 million individuals and killed 6.7 million people all over the world. On March 11, 2020, the World Health Organization (WHO) declared COVID-19 a worldwide epidemic, the novel coronavirus disease. COVID-19 is a fast-growing disease with inadequate hospital resources (WHO, 2022). During COVID-19, numerous molecular pathways (Saba et al., 2020) shown evidence of myocardial damage (Cau et al., 2021a), diabetes (Viswanathan et al., 2021), pulmonary embolism (Cau et al., 2021b), vascular damage (Khanna et al., 2022), and thrombosis (Fanni et al., 2021). Early, quick, and accurate identification of COVID-19 sickness is crucial to saving lives and protecting frontline workers due to the absence of a proper vaccine or medication. One of the gold standards for COVID-19 detection is RT-PCR, commonly known as “reverse transcription-polymerase chain reaction” (Gibson et al., 1996; Bustin et al., 2005). Furthermore, there is a need for new detection techniques due to the RT-PCR test’s slowness and low sensitivity (Fang et al., 2020). Because of superior sensitivity and repeatability in the diagnosis of COVID-19, imaging-based diagnosis such as chest X-ray (Nillmani et al., 2022), and computed tomography (CT) are becoming more popular in diagnosing and controlling COVID-19 infection (Sluimer et al., 2006; Saba and Suri, 2013; Giannitto et al., 2020; Cau et al., 2021c).

Healthcare imaging research and development have increased as a result of computer-aided diagnosis using machine learning (ML) (Suri and Rangayyan, 2006; Shrivastava et al., 2015) and artificial intelligence (AI) (Winston, 1992; Ramesh et al., 2004; Hamet and Tremblay, 2017). The potential benefit of AI to mimic manual delineation has speeded up the identification and diagnosis of illnesses (Molinari et al., 2007; Acharya et al., 2011, 2012a,b, 2013a,b,c; Pareek et al., 2013; Biswas et al., 2018a, 2019; Saba et al., 2019, 2021; Agarwal et al., 2021). AI techniques have tried to precisely duplicate the human brain using neural networks. This makes them capable of resolving imaging-related problems. Feature extraction, classification, and segmentation are all completely automated using deep layers in deep learning (DL), a subfield of AI (Ker et al., 2017; Litjens et al., 2017; Shen et al., 2017; Razzak et al., 2018; Fourcade and Khonsari, 2019; Hesamian et al., 2019; Zhou et al., 2019).

The primary imaging benefit of CT (Saba and Suri, 2013; Pathak et al., 2020; Wu X. et al., 2020) imaging is the ability to detect anomalies such as consolidation, ground-glass opacity (GGO) (Salehi et al., 2020; Cozzi et al., 2021), and other opacities that can be detected in the CT for a COVID-19 patient (Xie et al., 2020). Most chest CT lung scans frequently contain the GGO abnormality (Gozes et al., 2020; Yang et al., 2020; Shalbaf and Vafaeezadeh, 2021; Cau et al., 2021c). Most radiologists evaluate COVID-19 lesions using judgmental and semantic approaches due to time restraints and the vast volume of data. Additionally, the human and semi-automated evaluations take a lot of time, sluggish, and subjective (Alqudah et al., 2020; Xu et al., 2020; Aslan et al., 2021; Wu et al., 2021). As a result, to increase the timeliness of diagnosis for early COVID-19 sickness, rapid and error-free detection and real-time prognosis solutions are needed.

Several studies have been tried for COVID-19 lesion segmentation. They have been categorized into non-UNet-based solutions such as Ding et al. (2021), and UNet-based solutions (Hou et al., 2021; Lizzi et al., 2021; Paluru et al., 2021). A slight deviation from UNet was Generative Adversarial Network (GAN) by Zhang et al. (2020) and DR-MIL model by Qi et al. (2021). The challenges with these models were their low Dice Similarity Coefficient (DSC) in their prediction systems. Further, these techniques do leverage on the hybrid nature of the DL system design, nor there was an attempt to model them in the cloud-based framework or a reduction in the AI model size framework. A detailed analysis of previous methods will be discussed in a benchmarking subsection in the Discussion section.

To overcome the shortcomings of low DSC in the prediction, we proposed two HDL-based on UNet3+ framework. These models required less training data to achieve higher prediction scores. Further, we designed these HDL models in (a) a quantization framework for reduced model size and (b) in cloud-based settings. Thus, the following are the study’s primary contributions: (i) COVLIAS 3.0 was designed for the cloud and uses a quantized hybrid of Solo DL (SDL) and Hybrid DL (HDL) to target the lesion location for quicker segmentation. Annotations from one expert radiologist were used to train UNet3+ and two HDL models, namely VGG-UNet3+ and ResNet-UNet3+. (ii) A cohort of 3,500 CT scans chosen from a set of 45 COVID-19-positive patients for cross-validation using a 5-fold (K5) technique. (iii) A 500-image dataset that had never been seen before was used to validate the system. (iv) The computation of Area Error, Dice Similarity, Jaccard Index, Bland–Altman Plots, and Correlation Coefficient Plots comprised the performance evaluation systems. (v) Using quantization to reduce the storage space and prediction time of the final models. (vi) Statistical tests including the Mann–Whitney, paired *t*-test, Wilcoxon, and Freidman test, together with the *p* values, showed their stability and reliability. (vii) The online system took less than 1 s for each slice.

## 2 Methods

### 2.1 Demographics and baseline characteristics

**Figure 1:**
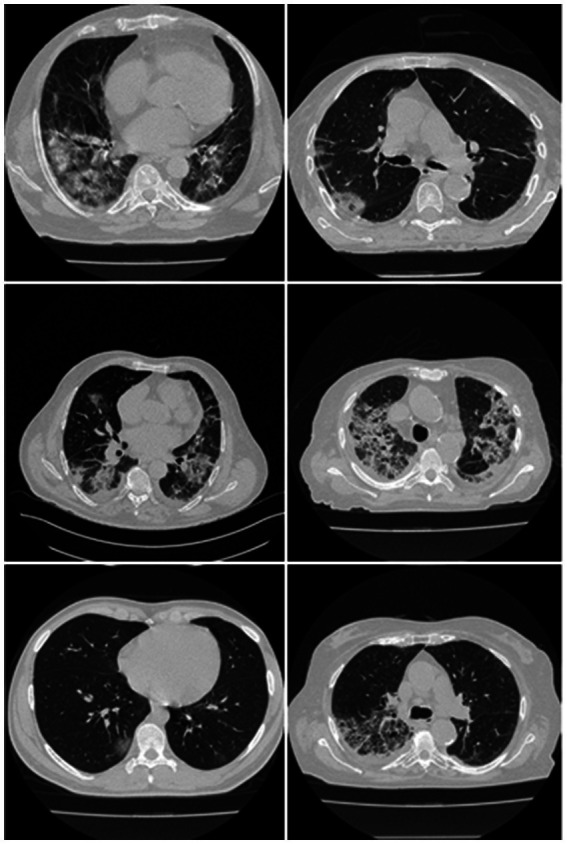
Raw CT images from the Croatian dataset.

The training cohort consisted of approximately 3,500 (3,542) CT images that were derived from 45 Croatian patients (Figure 1). With a mean age of 67, the patients were split into 37 men and the remaining females (SD 7.588). The group’s average GGO and consolidation scores were 2.5 and 1.5, respectively. In the cohort of 45 patients, all had coughs, 85.5% had dyspnea, 28% had hypertension, and 13.5% smoked, but none had cancer, diabetes, chronic obstructive pulmonary disease (COPD), or any other significant disorders. They did not all die from COVID-19 infection and were not all sent to the intensive care unit (ICU).

### 2.2 Image acquisition and data preparation

**Figure 2:**
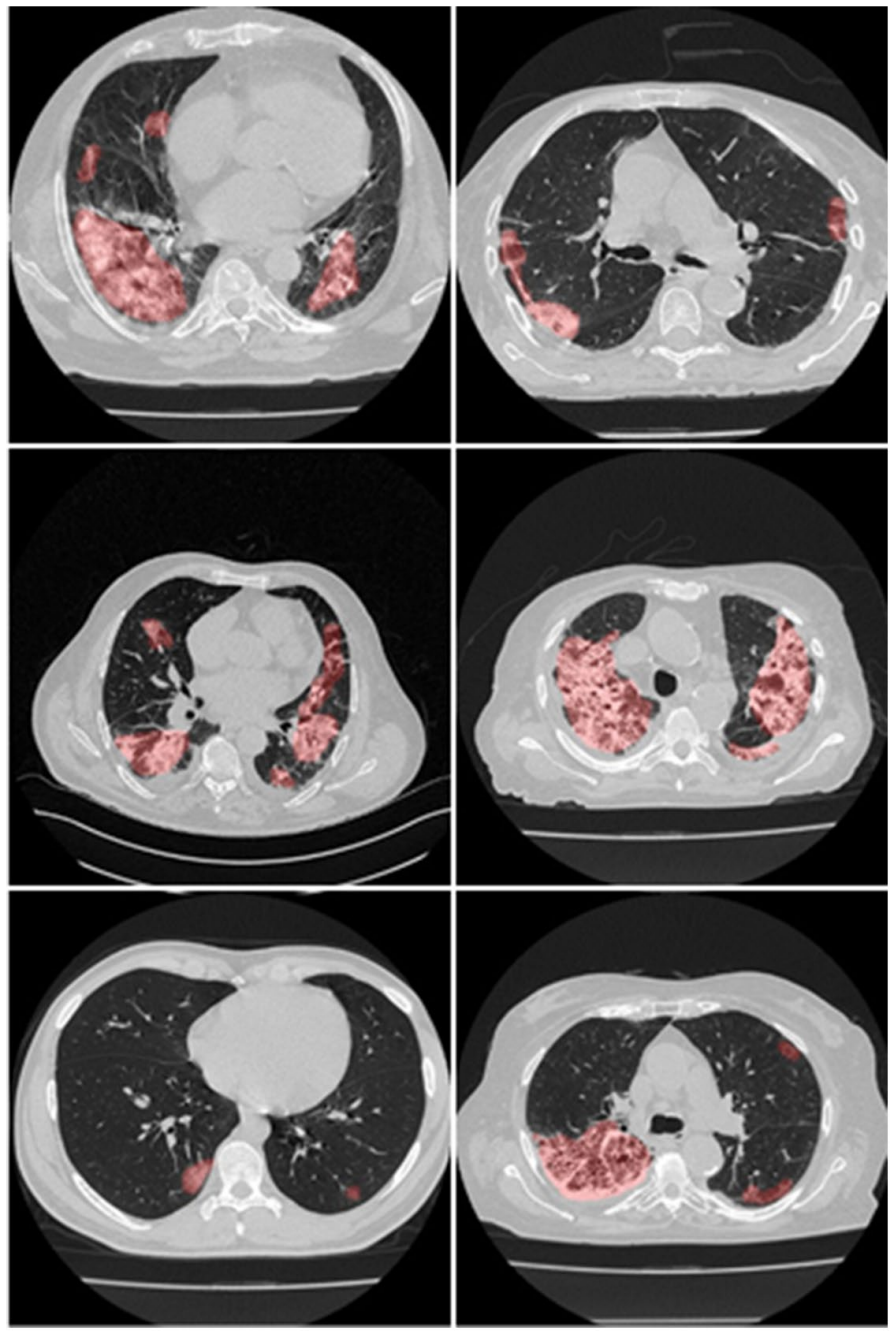
Manual overlays (red) on raw CT images.

UHID Ethics committee approved this study investigation where 45 COVID-19-positive Croatian cohorts were considered. The data were collected retrospectively between March 1 to December 31, 2020, at the University Hospital for Infectious Diseases in Zagreb, Croatia. The patient who met the following criteria: age > 18 years old, who had positive test results on RT-PCR, oxygen saturation 92% (hypoxia), respiratory rate 22/min (tachypnea), pulse rate > 100 (tachycardia), and systolic blood pressure 100 mm Hg (hypotension), went for thoracic MDCT scans. Fujifilm Corporation, Tokyo, Japan, 2017 vendor was used having the CT hardware 64-detector FCT Speedia HD. The technique used for CT acquisition was an inspiratory breath-hold (single) in the craniocaudal direction. System Software Version: V2.25, Copyright Hitachi, Ltd. 2017 had the following voltage and current ratings (120 kV, 350 mA having a rotation speed of 0.75 s). Using these parameters, standard Supria software was used for the whole-body X-ray CT imaging. The imaging parameters were: slice thickness of 1.25 mm along with recon index of 1 mm for picture filter 22 (lung standard). The iterative algorithm adopted was Intelli IP Lv.2 (WW1600/WL600). The criteria considered for imaging adopted reasonable image quality acceptance or no motion artifact due to patient movement and ensuring the presence of no metallic objects. The volume acquired consisted of ~300 slices, out of which ~70 CT slices (512 × 512 px^2^) were extracted by the senior radiologist, which accounted for about 23% of the total CT slice. The red color marked annotated lesion over the grayscale raw CT scan can be seen in Figure 2.

### 2.3 The deep learning models

To more quickly locate and segment lesions, the suggested study combines SDL and HDL models. The invention of merging two SDL models came about as a result of a recent demonstration that the combination of two HDL models, as opposed to the SDL models, had better feature extraction power (Jena et al., 2021). Therefore, two HDL models—namely, VGG-UNet3+ and ResNet-UNet3+ were used in this investigation. They were trained using data from a single expert radiologist and compared the SDL, namely, UNet3 + .

#### 2.3.1 The SDL: UNet3+

**Figure 3:**
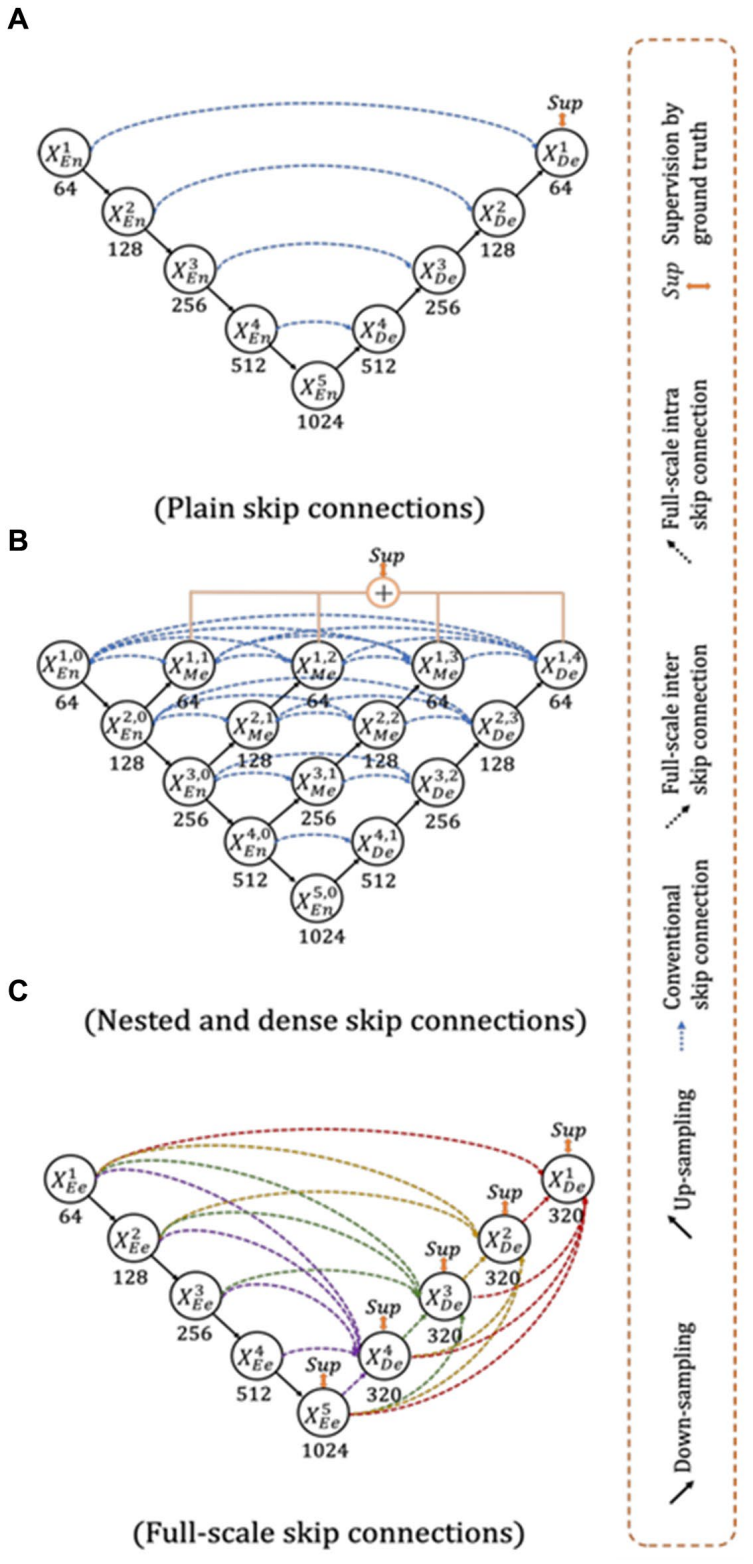
Top: UNet (Ronneberger et al., 2015), Middle UNet++ (Zhou et al., 2020), and Bottom: UNet 3+ (Huang et al., 2020).

The UNet3+ (Figure 3) were proposed by Huang et al. (2020) as a full scales-connected architecture designed for medical image segmentation. UNet3+ is a DL model that explores full-scale skip connections, unlike that of UNet++, which uses interlinked and dense skip connections, but refrains from full scales connections. The advantage of using full-scale skip connections over and above interlinked and dense skip connections is that the model combines low-level information from the images with high-level meanings from feature maps at different levels of resolution on the image. In contrast to the UNet, a collection of inter encoder-decode skip connections applies a non-overlapping max pooling operation to convey low-level detailed information from the smaller-scale encoder layers X^1^_En_ and X^2^_En_. Finally, to make the model understand the hierarchical features from the full-scale feature maps, full-scale deep supervision is also used. Note that this study does not implement UNet and UNet++; they are mentioned just to show how the new UNet3+ and its hybrid variants were derived.

#### 2.3.2 HDLs: VGG-UNet3+ and ResNet-UNet3+

The VGGNet architecture was designed to shorten training time by substituting 11 and 5-sized filters for the initial layer’s kernel filter (Simonyan and Zisserman, 2014). VGGNet was incredibly quick and efficient, but it struggled with optimization because of vanishing gradients. Because it is compounded by the gradient at each epoch and the update to the initial layers is so small, backpropagation produces far less training with no weights. To solve this problem, Residual Network, often known as ResNet (He et al., 2016), was developed. Gradients can now skip a select few layers in this design thanks to a new connection known as the “skip connection,” which solves the disappearing gradient problem. An identity function was also added to the network during the backpropagation step to maintain the local gradient values to a non-zero value.

By fusing one SDL (VGG or ResNet) with another SDL (UNet3+), the HDL models create a superior network that benefits from the strengths of both parent networks (Das et al., 2022). Three components make up the VGG-UNet3+ and ResNet-UNet3+ architectures used in this study: an encoder, a decoder, and a pixel-wise SoftMax classifier.

### 2.4 Loss function for SDL and HDL models

During the model creation process, the new models adopted the binary cross-entropy (BCE)-loss functions (Shore and Johnson, 1981; De Boer et al., 2005; Jamin and Humeau-Heurtier, 2019). The loss function can be mathematically described as given in Equation 1 if $\alpha_{BCE}$ represented the BCE-loss function, $a_{i}$ represented the classifier’s probability utilized in the AI model, x *_i_* represented the input gold standard label 1, (1− x *_i_*) represented the gold standard label 0.


(1) $$\alpha_{CE}=-\left[\left(x_{i}\times \log\;a_{i}\right)+\left(1-x_{i}\right)\times \log\left(1-a_{i}\right)\right]$$

Here × represents the product of the two terms.

The dice loss is named after the Dice-Sørensen coefficient, a statistic developed in the 1940s to evaluate the similarity between two samples. It was introduced to the computer vision field by Milletari et al. (2016) for the segmentation of 3D medical images. When *X* is the input image and *Y* is the target or ground truth image, the Dice loss (D) employed in this manuscript can be represented as given in Equation 2.


(2) $$D=1-\frac{2\left|X\cap Y\right|}{\left|X\right|+\left|Y\right|}$$

### 2.5 Experimental protocol

Standardized cross-validation (CV) method was used to assess the accuracy of the AI models. Our team has developed several CV-based protocols of various types for a variety of applications using the AI framework (Acharya et al., 2013b; Shrivastava et al., 2015; Araki et al., 2016; Maniruzzaman et al., 2018). We employed the K5 cross-validation methodology using observed data analysis, consisting of 80% training (2,800 scans) and 20% training data (700 CT scans). Because of the favorable COVID-19 parameters, the 5-fold was chosen. Here, in each fold, the chance was given to have its own test set, where 10% of the data was taken into consideration for protocol’s internal validation mechanism. The test data consisted of unseen 500 COVID-19 positive images for generalizability.

The accuracy (ACC) of the AI system is assessed by contrasting predicted output with actual ground truth pixel values. The black and white pixels of the output mask were converted to binary 0/1 integers for further processing. Using the standardized symbols TP, TN, FN, and FP to signify true positive, true negative, false negative, and false positive, truth table was designed for accuracy determination (Eq. 3).


(3) $$ACC\;\left(\%\right)=\left(\frac{TP+TN}{TP+FN+TN+FP}\right)\times 100$$

## 3 Results and performance evaluation

### 3.1 Results

**Figure 4:**
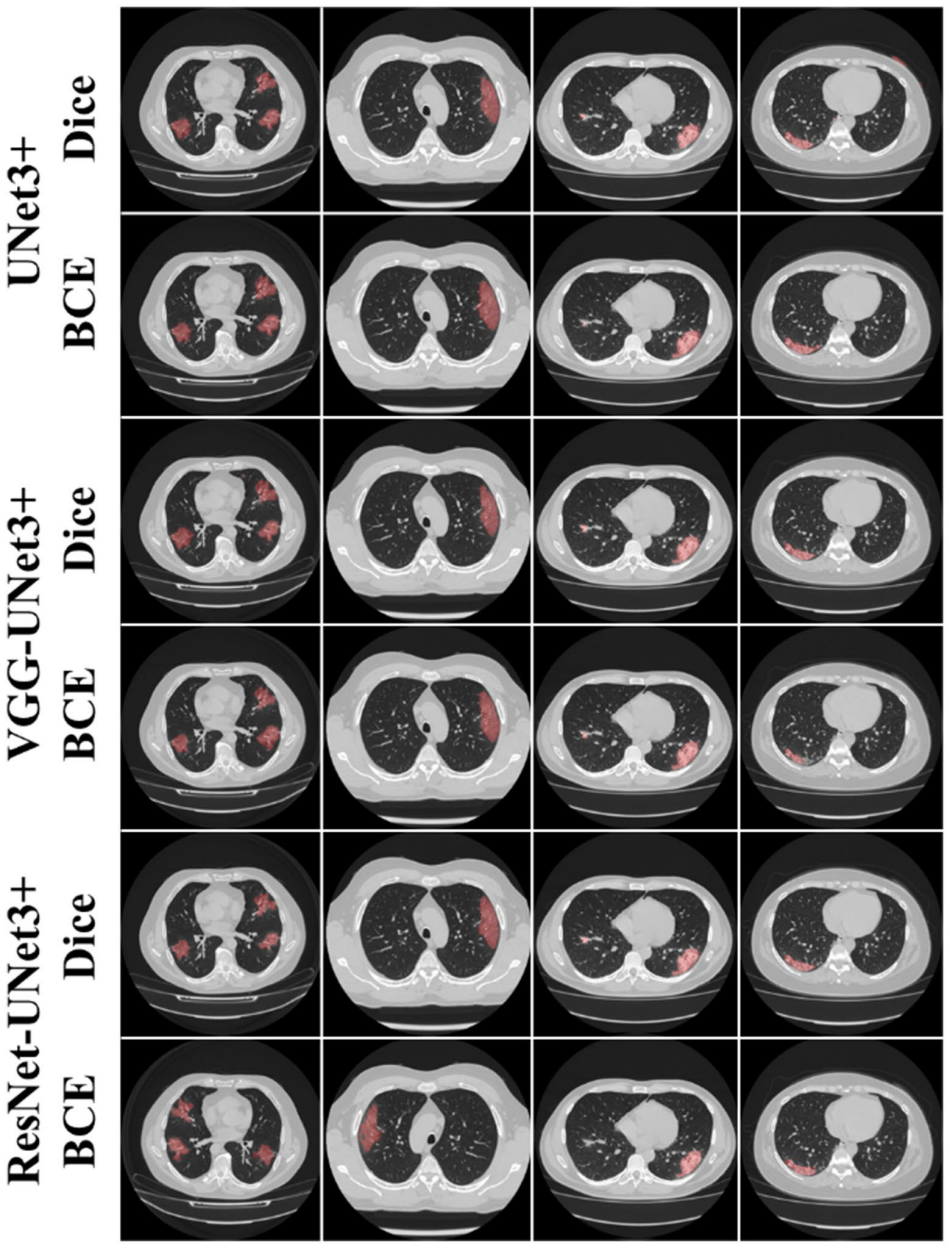
AI predicted COVID-19 lesion overlay, using three models: UNet3+ (row 1 and 2), VGG-UNet3+ (row 3 and 4), and ResNet-UNet3+ (row 5 and 6). BCE and Dice are the two loss functions.

This proposed study is a novel implementation of two HDL architectures VGG-UNet3+ and ResNet-UNet3+ for COVID-19-based lesion segmentation. A cohort of 3,500 lung CT images from 45 COVID-19 positive patients has been utilized with a five-fold CV technique. Another cohort of 500 COVID-19 positive patients from the MosMed (Russia) dataset was used as part of an unseen-AI analysis. Figure 4 shows the overlay of the DL predicted lesion using the three DL models UNet3+, VGG-UNet3+, and ResNet-UNet3+ for the dice and BCE loss functions, using the unseen dataset.

### 3.2 Performance evaluation

**Figure 5:**
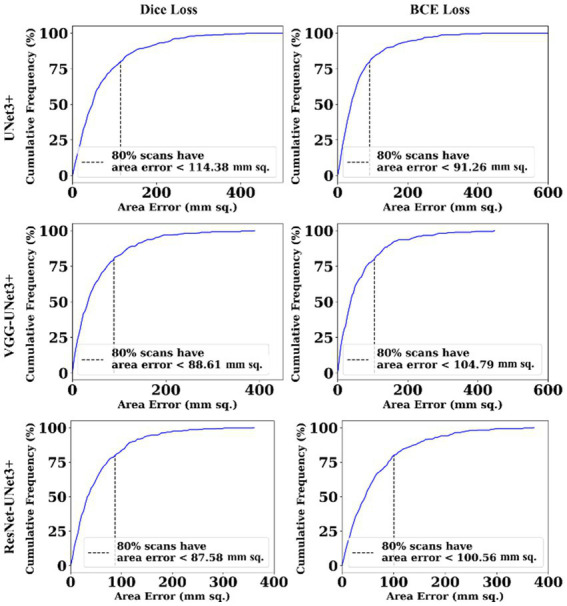
Area error.

**Figure 6:**
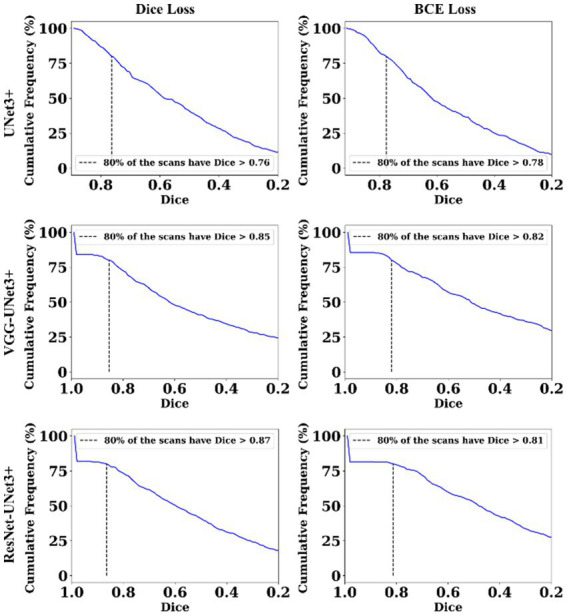
Dice Similarity plot.

**Figure 7:**
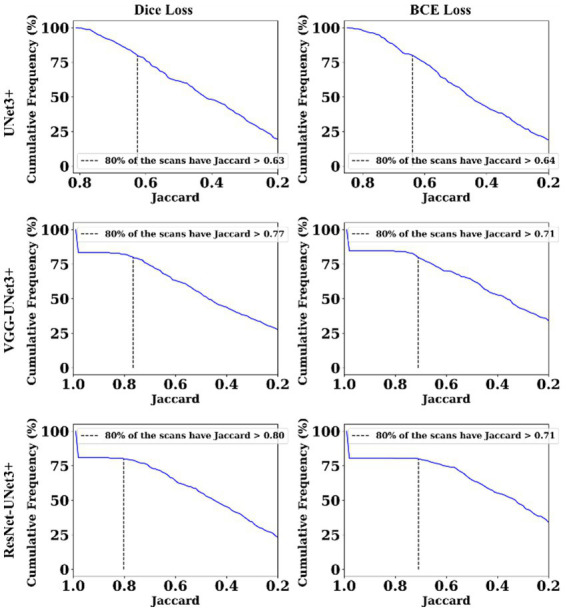
Jaccard Index plot.

**Figure 8:**
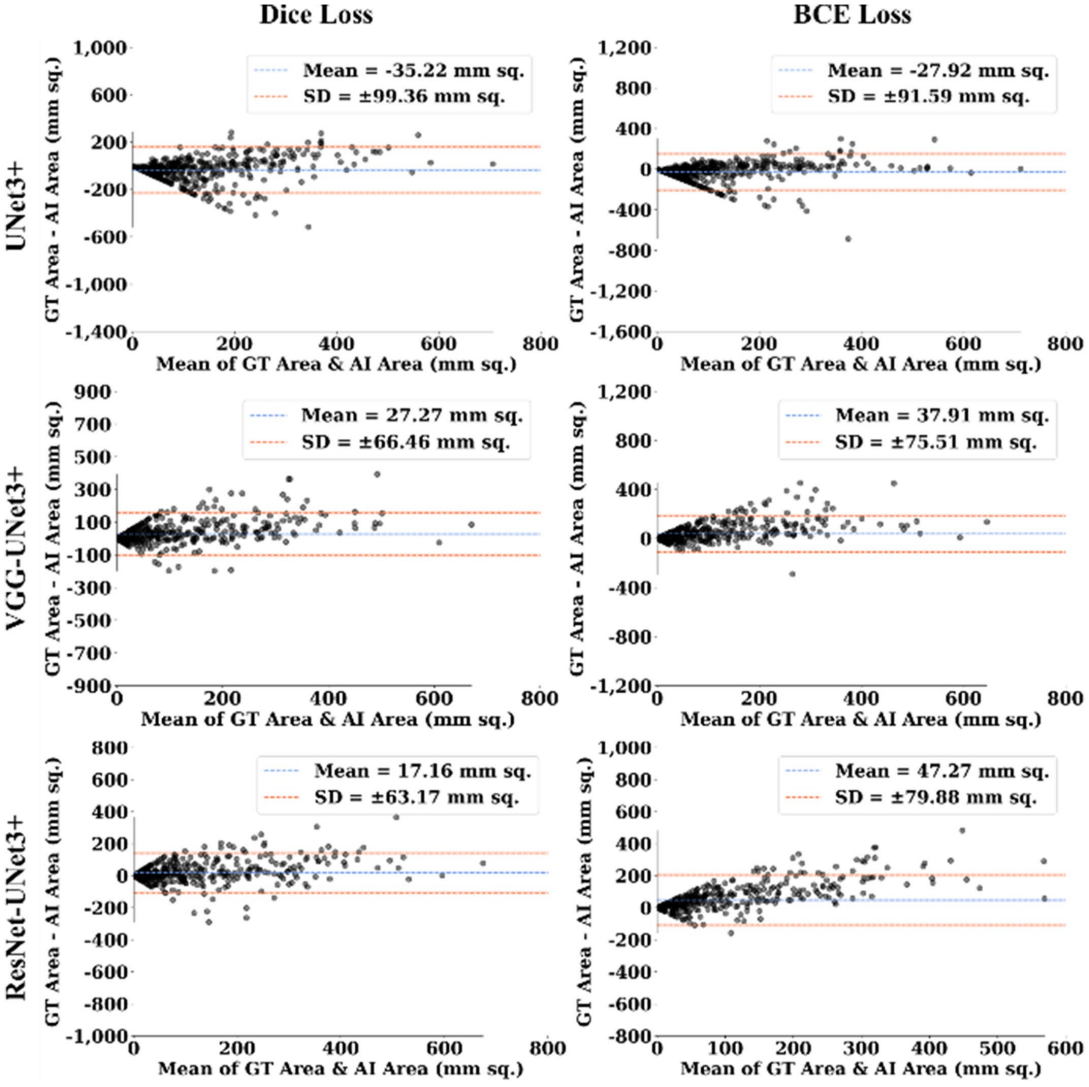
BA Plot.

**Figure 9:**
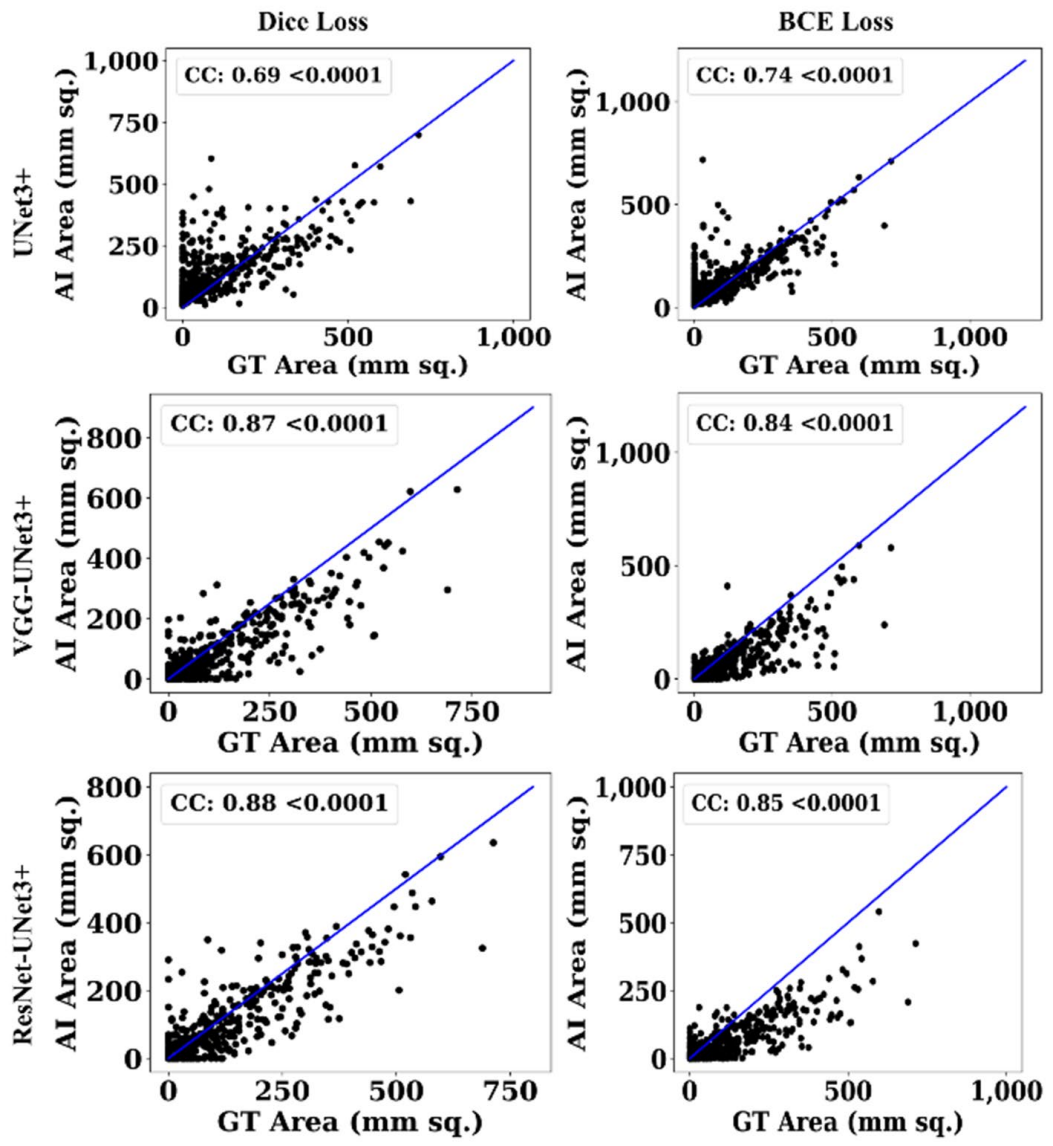
CC Plot.

This proposed study uses (i) Area Error (AE), (ii) Dice similarity (DS) (Basar et al., 2022; Chu et al., 2022), (iii) Jaccard index (JI) (Eelbode et al., 2020), (iv) Bland–Altman (BA) plots (Dewitte et al., 2002; Giavarina, 2015), and (v) Correlation coefficient plots, for the three DL models against Dice and BCE loss for performance evaluation, using the unseen dataset containing 500 CT images. Figures 5–7 show the cumulative frequency distribution (CFD) plot for Area error, DS, and JI for UNet3+, VGG-UNet3+, and ResNet-UNet3+, respectively, and depicts the score at an 80% threshold. Figures 8, 9 depict the BA and CC plots for the three DL models. This study also uses manual delineation from a trained radiologist to validate the results from the three DL models, thus, useable for clinical settings. Using the performance evaluation on the unseen dataset, the HDL model ResNet-UNet3+ outperformed all the other models proposed in this study, thereby proving the performance of the HDL model is superior to the SDL model.

## 4 Discussion

The main application domain is the pulmonary field of medicine combined with radiological imaging which involves AI-based solution for segmentation of COVID-19 lung lesions embedded with pruning framework in cloud-based settings. This study uses one SDL UNet3+ and two HDL models, (i) VGG-UNet3+ and (ii) ResNet-UNet3+, trained using a 5-fold cross-validation technique utilizing a set of 3,500 manually annotated images, to demonstrate automatic lesion identification in a DL framework. The three DL models in this proposed study are trained using Dice and BCE loss and tested against the unseen dataset of 500 CT images utilizing (i) AE, (ii) DS, (iii) JI, (iv) BA, and (v) CC plots. Considering these metrics, the best AI model, ResNet-UNet3+ was superior to UNet3+ by 17 and 10% using Dice loss and BCE loss when compared against a seen dataset. Thereby establishing that the dice performed better than BCE loss for COVID-19 lesion segmentation. Further, the COVLIAS 3.0 showed DSC was 16% better when comparing against the mean DSC of previous studies (Zhang et al., 2020; Ding et al., 2021; Lizzi et al., 2021; Paluru et al., 2021). Mann–Whitney, Paired t-Test, Wilcoxon, and Friedman tests demonstrated the stability and scientific reliability of the proposed system, with a *p* value <0.001 (Table 1). To speed-up the training process NVIDIA’s DGX V100, with multi-GPU, was adopted. The results show that ResNet-UNet3+ is the best model out of all the DL models.

**Table 1:** Statistical test.

	Dice loss			BCE loss		
Model	UNet3+	VGG-UNet3+	ResNet-UNet3+	UNet3+	VGG-UNet3+	ResNet-UNet3+
Paired *t*-test	*p* < 0.001	*p* < 0.001	*p* < 0.001	*p* < 0.001	*p* < 0.001	*p* < 0.001
Mann–Whitney	*p* < 0.001	*p* < 0.001	*p* < 0.001	*p* < 0.001	*p* < 0.001	*p* < 0.001
Wilcoxon	*p* < 0.001	*p* < 0.001	*p* < 0.001	*p* < 0.001	*p* < 0.001	*p* < 0.001
Friedman test	*p* < 0.001	*p* < 0.001	*p* < 0.001	*p* < 0.001	*p* < 0.001	*p* < 0.001

Table 2 lists the key metrics for comparing the three models, describing (i) the loss function used while training, (ii) the total number of AI model parameters, (iii) the number of layers, (iv) the size of the final saved model, (v) the number of training epochs, (vi) the batch size, and (vii) the online prediction time per image for COVLIAS 3.0.

**Table 2:** Model parameters.

SN	Attributes	UNet3+	VGG-UNet3+	ResNet-UNet3+
1	Loss function	BCE & Dice		
2	# Parameters	~26 M	~20 M	~15 M
3	# Layers	114	81	157
4	Size (MB)	299	125	106
5	# Epoch	50	50	50
6	Batch size	4	8	8
7	Prediction time	~2 s	~1 s	~1 s

M, Million; MB, Mega Byte; #, Number.

### 4.1 A short note on cloud-based COVLIAS 3.0

In cloud-based setting, the patient and physician relationship can be very efficient, especially during the virology period. In all such setups, it is vital to demonstrate the usage of the visual images. These visual images carry a deep role especially under explainability paradigm. The trust of the physicians to use the software system is the most important component in medical imaging. This was demonstrated in our previous contributions (Saba et al., 2016, 2017, 2018, 2023). Some of these applications are in cardiology applications. These visual displays serve two purposes: (i) show the comprehension nature of the design depicting and (ii) proves the nature of explainability. Both these objectives are met in our display. Further, the figures provide an overview of the system and display the overall pipeline of the system. To make the system accessible, we have made a web-based AI system using Amazon Web Services. The system is capable of processing single as well as multiple CT images at a time. After selection of the image(s), the system loads the AI model and segments the COVID-19 lesion and displays it as a report, which can be downloaded as a portable document format (PDF).

**Figure 10:**
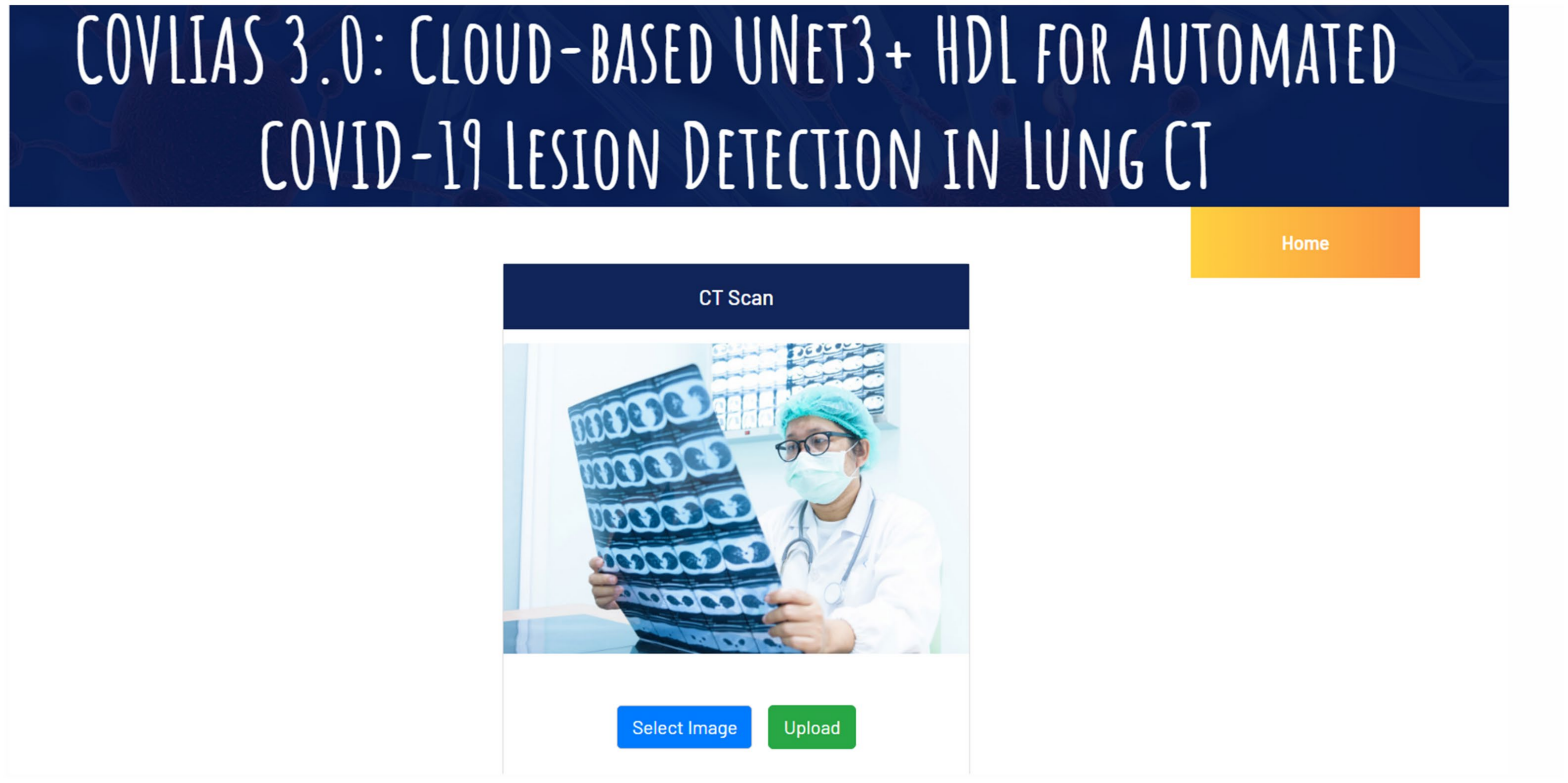
Landing page of COVLIAS 3.0: Hybrid AI-Based COVID-19 Lesion Segmentation system.

**Figure 11:**
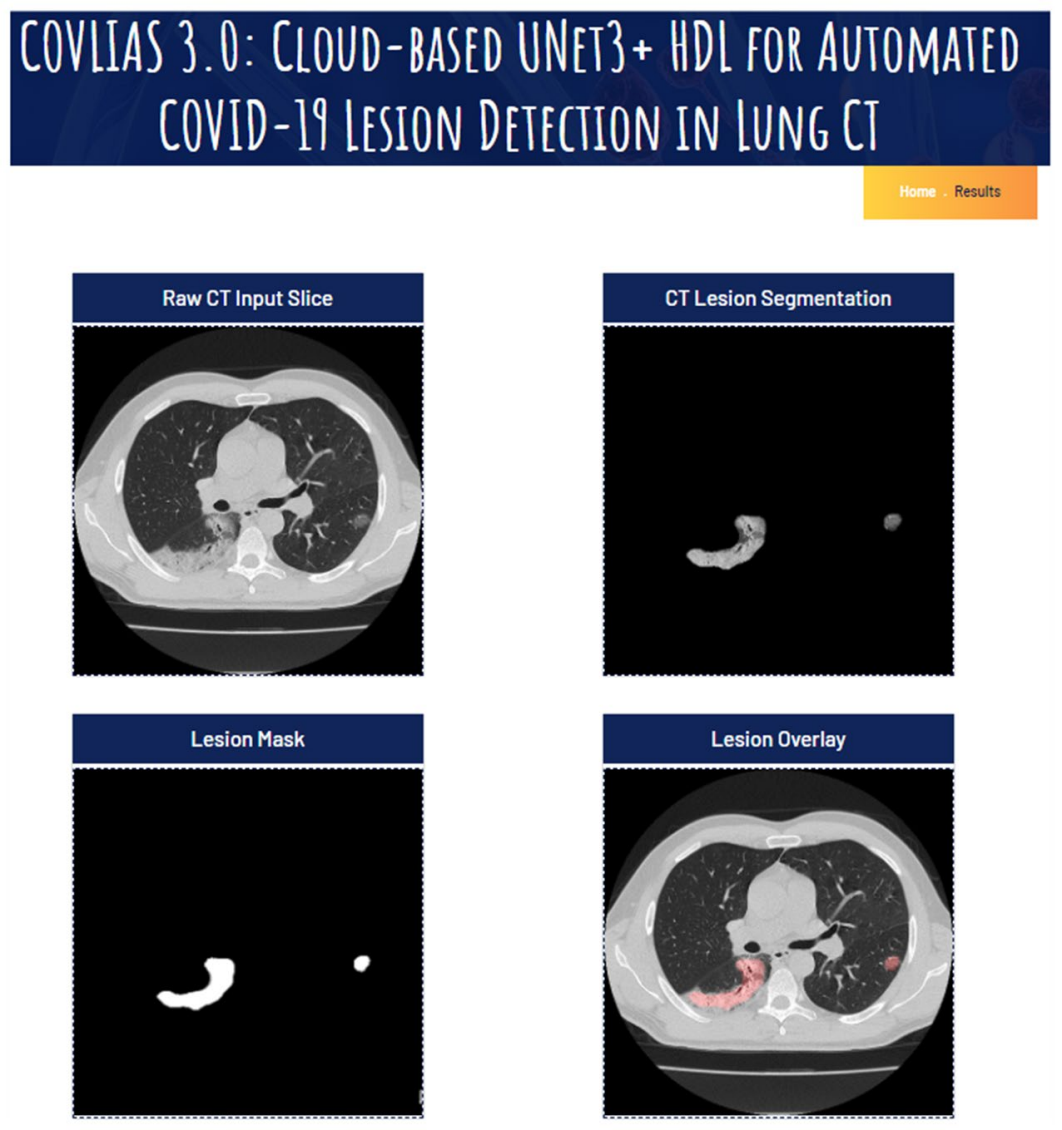
Snapshot of the result page using COVLIAS 3.0: Hybrid AI-Based COVID-19 Lesion Segmentation system.

Note that a certain pre-processing discussed in our previous research (Suri et al., 2022a,b) must be done before the system can accept the image and process it for segmentation and analysis. Each run is assigned a unique identification ID, which allows it to be easily accessible for analysis purposes and can also be used to finetune the AI model at a later stage. Figure 10 represents the landing page of COVLIAS 3.0: Hybrid AI-Based COVID-19 Lesion Segmentation system. Figure 11 presents the output from the web-based COVLIAS 3.0 system. To make the cloud deployment cost-effective and reduce the processing time, we have utilized multiprocessing and load-balancing.

### 4.2 Quantization

Quantization in deep learning is the process of reducing the number of bits used to represent the weights and parameters of a neural network model (Wu H. et al., 2020; Ma H. et al., 2021). By reducing the precision of the parameters, the overall size of the model can be dramatically reduced, which can have a significant impact on the speed of computation and the amount of memory required. In other words, it is the process of reducing the resolution of a pixel by reducing the number of its possible values. This is typically done by rounding off the pixel’s values to a predetermined set of values, which are referred to as levels.

Additionally, quantization can also be used to improve the accuracy of the model by reducing the impact of noise and other distortions. Quantization is used in many different areas of signal processing, including digital audio, image processing, and communication systems.

#### 4.2.1 Advantages of quantization in deep learning

(1) *Reduced memory and storage requirements*: By using fewer bits to represent the weights and parameters of the model, the overall size of the model is reduced, which can significantly reduce the memory and storage requirements. This can be especially beneficial for deploying models to devices with limited memory and storage capabilities. (2) *Improved model performance*: Quantization can also lead to improved model accuracy and performance by reducing the impact of noise and other distortions. (3) *Faster computation times*: By reducing the precision of the parameters, the computational complexity of the model is reduced, which can lead to faster computation times.

Table 3 presents a list of metrics for comparing the three quantized models, describing (i) the size of the final saved model, (ii) the size of the final quantized (compressed) saved model, (iii) percentage (%) size reduction, and (iv) the online prediction time per image for COVLIAS 3.0 using quantized models. Quantized UNet3+, VGG-UNet3+, and ResNet-UNet3+ models were able to achieve 66.76, 36.64, and 46.23% compression, respectively.

**Table 3:** Quantization.

SN	Attributes	UNet3+	VGG-UNet3+	ResNet-UNet3+
1	Size (MB)	299	125	106
2	Compressed size (MB)	99.4	79.2	57
3	% Size reduction	66.76%	36.64%	46.23%
4	Prediction time	<1 s	<0.5 s	<0.5 s

MB, Mega byte.

### 4.3 Benchmarking

With Res2Net50 (Gao et al., 2019) as its foundation, Ding et al. (2021) developed MT-nCov-Net, a multitasking DL network that comprised the segmentation of both lesions and lungs in CT images. More than 36,000 scans from five separate CT imaging databases were used in this investigation. The study adopted random flipping, rotation, cropping, and Gaussian blurring as part of the augmentation protocol, resulting a Dice of 0.86. Hou et al. (2021) used an improvised canny edge detector (Ding and Goshtasby, 2001; McIlhagga, 2011) on CT scans to identify COVID-19 lesions. The authors used a dataset of roughly 800 CT images. Lizzi et al. (2021) designed a cascaded UNet system for COVID-19-based lesion segmentation on CT images, using a variety of augmentation methods, including zooming, rotation, Gaussian noise, elastic deformation, and motion blur, were applied. The authors showed a DSC of 0.62, compared to Ding et al. (2021)'s value of 0.86. The network DR-MIL shown by Qi et al. (2021) was built on the foundation of ResNet-50 and XceptionNet (Chollet, 2017). In this work, rotation, reflection, and translation were applied as image augmentation techniques on around 2,400 CT scans. The study did not mention about DSC. Paluru et al. (2021) introduced Anam-Net, a hybrid of UNet and ENet. This method required an additional step of lung segmentation prior to COVID-19 lesion segmentation. While using a training cohort of 4,300 CT scans, the system showed DSC of 0.77. The Anam-Net system was designed for Android application on an edge device. Zhang et al. (2020) introduced CoSinGAN, a GAN network for COVID-19-based lesion segmentation. This GAN employed only 700 CT lung images for training and used no augmentation. The DSC using CoSinGAN’s was 0.75. Cai et al. (2020) used the UNet-based model and adopted a 10-fold CV protocol on 250 images and showed a DSC of 0.77. Using the same methodology, the author presented lung and lesion segmentation. The length of an intensive care unit (ICU) stay can be predicted by the authors using the results of lesion segmentation. For 3D CT volume segmentation, Ma J. et al. (2021) also applied the typical UNet design to a collection of 70 patients. The training phase also included model optimization, and the study reported a DSC of 0.67. The model’s performance was compared to that of other studies in the same field by the authors. Kuchana et al. (2021) employed UNet and Attention UNet to segment the lung and lesions in a group of 50 patients. The model showed a DSC of 0.84 after the authors improved the hyperparameters during the training procedure.

Finally, Arunachalam et al. (2021) presented a two-stage lesion segmentation technique, where, stage-I involved employing region-based convolutional neural networks (RCNN) to estimate the region of interest, while stage-II involved creating bounding boxes. For the train, validation, and test sets, the performance metrics were 0.99, 0.931, and 0.8. In conjunction with automated bounding box estimates for mask production, the RCNN was predominantly used for COVID-19 lesion identification. Our COVLIAS 3.0 leverages hybrid model technology demonstrating a higher DSC of 16% and further implemented in cloud-based framework embedded with quantization infrastructure thereby reducing the training model sizes.

### 4.4 Strengths, weakness, and extensions

UNet has been evolving over the last 7 years, especially in ultrasound (Sanches et al., 2012; Jain et al., 2021, 2022). The modifications to fundamental UNet have given the power to the segmentation process, which includes the addition of more stochastic image processing techniques in UNet framework (El-Baz et al., 2011, 2015; Shrivastava et al., 2015). Using the hybrid system with advanced UNet that uses full-scale skip connections has improved the performance of the system. Further, quantizing the DL models helped reduce the storage space and overall computation time in the cloud framework.

The following are the two main limitations: (i) The major weakness of the above model is the lack of strong features extraction or small feature extraction. The addition of attention models or transformer models can improve this. (ii) Another limitation of this protocol is the requirement of hardware such as graphical processing unit (GPU). Since GPUs are not easily available in all universities, this can be a limitation when applying pruning-based segmentation models.

#### 4.4.1 Implications

Small features can be detected by adding attention-based models. Multithreaded architectures can be used for increasing the speed of the system. Empirical convergence can be used during the training process, which involves fixed number of epochs where the validation loss can be close to training loss. Further, we can converge by taking training number of epochs less than a threshold value.

Due to recent advances in pruning models (Agarwal et al., 2022) and evolutionary methods, one can extend this to the UNet framework. In the future, more variants of hybrid systems (DL with ML) can be used and tested for the performance and reliability of the system (Biswas et al., 2018b). To make the system more robust, the system can be trained on a combination of the dataset from different countries, ethnicities, patients with comorbidities as tried in other modalities (Skandha et al., 2022). There are other potential applications of such technologies that are not limited to mammography, urology, pulmonary, ophthalmology, neurology, nephrology, and cardiology. This includes diagnosis of lesions in brain, breast, prostate, retinal, renal, lung, and heart.

## 5 Conclusion

To handle the lesion localization and segmentation more quickly, the proposed study provides three DL models for lesion segmentation in 3,500 CT images (Croatia) obtained from 45 COVID-19 patients. One experienced radiologist was used to train the one SDL namely UNet3+, and two HDL models, namely, VGG-UNet3+, and ResNet-UNet3+. For performance evaluation, the training program used a 5-fold cross-validation technique. It makes use of tracings from two qualified radiologists as part of the validation. Using the unseen dataset of 500 CT images and the AE, DS, JI, BA, and CC plots, the three DL models in this proposed study were evaluated against Dice and BCE loss.

The key takeaway was that ResNet-UNet3+ was superior to UNet3+ by 17 and 10% for Dice and BCE loss when compared against an unseen dataset. Second takeaway was that the Quantized UNet3+, VGG-UNet3+, and ResNet-UNet3+ models were able to achieve 66.76, 36.64, and 46.23% compression, respectively. The third takeaway was that since the system was designed for cloud-based settings. To sum up, our pilot research showed how consistently the HDL model could find and segment COVID-19 lesions in CT images superior performance.

## Data availability statement

The original contributions presented in the study are included in the article/supplementary material; further inquiries can be directed to the corresponding authors.

## Ethics statement

The use of artificial intelligence for multislice computer tomography (MSCT) images in patients with adult respiratory diseases syndrome and COVID-19 pneumonia (approval Code: 01-2239-1-2020) approval: authorized by the University Hospital for Infectious Diseases Dr. Fran Mihaljevic, Zegreb, Mirogojska 8. On November 9th, 2020. Approved to Klaudija Viskovic, MD, PhD. The requirement of written informed consent for participation was waived by the institutional review boards due to the retrospective nature of the research.

## Author contributions

SA: Data curation, Methodology, Software, Visualization, Writing – original draft. SS: Validation, Writing – review & editing. AC: Data curation, Validation, Writing – review & editing. GC: Validation, Writing – review & editing, Data curation. GR: Validation, Writing – review & editing, Funding acquisition. SP: Validation, Writing – review & editing. JL: Validation, Writing – review & editing. DG: Validation, Writing – review & editing. MFa: Writing – review & editing, Validation. LM: Writing – review & editing. AD: Writing – review & editing, Visualization. RS: Writing – review & editing. MFo: Writing – review & editing, Funding acquisition. NS: Writing – review & editing. SN: Validation, Writing – review & editing, Supervision. KV: Writing – review & editing, Data curation, Formal analysis, Validation. MKu: Investigation, Writing – review & editing, Validation. MKa: Supervision, Writing – review & editing. LS: Formal analysis, Investigation, Methodology, Supervision, Validation, Writing – review & editing. JS: Formal analysis, Investigation, Methodology, Resources, Supervision, Writing – review & editing.
